# Adenomyosis and *in vitro* fertilization impacts - A literature review

**DOI:** 10.5935/1518-0557.20200104

**Published:** 2021

**Authors:** Ana Luíza Assin Squillace, Daniela Simões Simonian, Marcella Cardoso Allegro, Edson Borges Júnior, Paulo Homem de Mello Bianchi, Mauro Bibancos

**Affiliations:** 1 Department of Urology, Pontifical Catholic University of Campinas, Campinas (SP), Brazil; 2 Fertility Medical Group, São Paulo (SP), Brazil; 3 Department of Human Reproduction. Medicine School. University of São Paulo; 4 Fivmed Laboratory, Department of Andrology, Fivmed Reproductive Medicine, Campinas (SP), Brazil

**Keywords:** Adenomyosis, endometriosis, infertility, *In Vitro* fertilization, embryo implantation

## Abstract

Adenomyosis is a gynecological condition, which is characterized by stromal and glandular endometrial tissue infiltration inti the myometrium, resulting in an increase of uterine volume. The etiology of adenomyosis is presently unknown, but some theories assist us in understanding its pathogenesis and natural history. Clinical manifestations are increased menstrual flow and dysmenorrhea, abnormal uterine bleeding, chronic pelvic pain, and dyspareunia. The signs and symptoms suggestive of adenomyosis are complemented by diagnostic methods such as transvaginal ultrasound (TVUS), ideally with intestinal preparation, magnetic resonance imaging (MRI) and surgery; although currently, there are no precise criteria for the classification of findings on imaging studies. The clinical and surgical therapeutic approach must be individualized, taking into account the patient’s characteristics, for instance, age, parity, depth and number of adenomyotic foci, uterine volume and, mainly, clinical manifestations. A causal relation between adenomyosis and infertility has been repeatedly suggested, mostly due to the anatomo-physiopathological conditions originated by the adenomyosis on the female genital tract; however, definitive conclusions are still lacking. This pathology is found in approximately 25% of infertile women, especially those who have had recurrent pregnancy loss (RPL), recurrent implantation failure, older women seeking In Vitro fertilization (IVF) treatment, and those with concomitant endometriosis. To determine whether adenomyosis per se affects fertility, several researchers have focused on women who are affected by the condition and underwent IVF/intracytoplasmic sperm injection (ICSI); for this model provides more accurate data about the influence of adenomyosis on embryo implantation. Therefore, our objective was to analyze, through a systematic literature review, the effect of uterine adenomyosis on the probability of pregnancy by IVF / ICSI, as well as trying to point out the main difficulties and gaps to establish a standard protocol for the management of these patients, since most of the patients with adenomyosis have other associated gynecological pathologies, mostly endometriosis; in addition to the heterogeneity of the studies still remaining as an obstacle to precise conclusions.

## INTRODUCTION

Over the past few years, many advances in knowledge and diagnostic methods related to infertility occurred and, consequently, technologies for assisted reproduction and embryonic analysis advanced. However, the way in which gynecological pathologies affect reproductive outcomes is not yet fully understood. Therefore, many studies have evaluated the impact of adenomyosis alone, and associated with endometriosis on fertility and IVF outcomes, but the data are inconsistent. That being the case, we considered it would be interesting to conduct a systematic literature review of the data published so far, in order to define more precisely the effect of uterine adenomyosis on the probability of pregnancy by IVF/ICSI, along with trying to point out the main difficulties and gaps to establish a standard protocol to manage these patients.

Adenomyosis is a gynecological condition, which is characterized by stromal and glandular endometrial tissue infiltration into the myometrium, causing an increase in uterine volume. The etiology of adenomyosis is presently unknown, but some theories assist us in understanding its pathogenesis and natural history, such as the invasion of the myometrium by endometrial tissue theory, and the migration of endometrial cells due to menstrual reflux theory. Risk factors such as prolonged exposure to estrogen, multiparity and uterine trauma are directly related to the theories proposed regarding the etiology of adenomyosis. It is known that its prevalence is circa 8 to 27%, with a higher prevalence in women in their last reproductive age decades, with pregnancy losses and recurrent implantation failure with assisted reproduction methods (Campo et al., 2012; Tomassetti, 2013; Mahajan et al., 2018). Clinical manifestations, for instance, an important increase in menstrual flow and dysmenorrhea, are reported by 65% of the women with adenomyosis, in addition to abnormal uterine bleeding, chronic pelvic pain and, less frequently, dyspareunia. However, most patients are asymptomatic. Many women, even those with the classic clinical signs of adenomyosis, will only face the pathology during conception. Therefore, more and more patients with adenomyosis require assistance to become pregnant, since the pathology may be directly linked with infertility and worse results in assisted reproduction treatments (Campo et al., 2012; Mahajan et al., 2018). Clinical signs and symptoms suggestive of adenomyosis are complemented by imaging methods, such as two-dimensional and three-dimensional transvaginal ultrasonography (TVUS), and pelvic magnetic resonance imaging (MRI), which help detect the pathology and differentiate it from other gynecological diseases. However, although imaging methods are of great relevance for the suspicion of the pathology, adenomyosis may only be diagnosed with the evaluation of myometrial tissue samples that show endometrial invasion, which makes the diagnosis difficult in women who still want to gestate. Accordingly, the clinical and surgical therapeutic approach must be individualized, taking into account the patient’s characteristics; for instance, age, parity, depth and number of adenomyotic foci, uterine volume and, mainly, clinical manifestations. The pharmacological therapy consists of the administration of non-steroidal anti-inflammatory drugs (NSAIDs), progestin or combined oral contraceptives, GnRH agonists, selective progesterone receptor modulators (SPRMs), steroidogenesis enzyme inhibitors, among others. Surgical methods, such as hysteroscopic endometrial ablation and resection, can be used as a basis to treat the adenomyosis. Other procedures used are uterine artery embolization, and laparoscopic or hysteroscopic electrocoagulation of the myometrium, which promotes the reduction of adenomyotic foci caused by necrosis. As a definitive treatment, hysterectomy is the first option for patients who have already completed their reproductive cycle and have severe symptoms.

As elucidated above, adenomyosis may present itself in several clinical and radiological forms, with findings suggestive of the condition. However, most patients who may have adenomyosis are asymptomatic, being one of the possible diagnoses when the physician and the couple are faced with infertility. (Lin et al., 2000)

## ADENOMYOSIS AND INFERTILITY

### Pathophysiological aspects

Theories advocate that the anatomo-physiopathological conditions generated by adenomyosis in the female genital tract could be related to infertility. Adenomyosis is found in 24.4% of infertile women, especially in those who suffered recurrent miscarriages and recurrent implantation failures, in older women seeking IVF treatment and in those with endometriosis (Puente et al., 2016; Sharma et al., 2019). The causal relation between adenomyosis and infertility has not yet been fully established, and currently, several studies attempt to list mechanisms that would justify this association.

Adenomyomas, principally submucosal and intramural, which anatomically alter the endometrial cavity, could, according to theory, obstruct the tubal ostium, consequently interfering on sperm and embryo migration (Kissler et al., 2006).

Another studied mechanism is the alteration of archimyometrium (myometrial part of the junctional zone) peristalsis, associated with a worse reproductive outcome. Myometrial contractions that, in excess, would affect the very etiology of adenomyosis, originate in the junctional zone. Under normal circumstances, junctional zone peristalsis is essential to transport sperm to the ipsilateral tubal ostium and to the dominant follicle (Kissler et al., 2006; Harada et al., 2016). Further, the endometrial invaginations of the junctional zone, in adenomyosis carriers, negatively affect this mechanism, seeing that their hypertrophied muscle fibers promote a marked peristalsis and increased endometrial pressure. In this case, dysperistalsis also impairs the successful outcome of IVF (Harada et al., 2016).

The impact of adenomyosis on female fertility could even be originated at a histological level. Mehasseb et al. (2011), concluded, after studying microscopically the uterine cells of patients with and without adenomyosis, that the myocytes in uterine adenomyosis appear to be structurally different when compared to normal myocytes. According to them, cells from patients with adenomyosis are more hypertrophic, with irregular sized nuclei and mitochondria, and have excessive myelin deposits, which would cause disturbance in the rhythm of myometrial contraction, also affecting utero-tubal sperm transport (Harada et al., 2016).

Going further into the endometrial cavity, another mechanism pointed out for the worst initial obstetric outcome would be the excessive inflammation present in the adenomyotic cavity when compared to the healthy one. Chronic inflammation is considered another theory for infertility; the mediating cell being local macrophages, which, producing pro-inflammatory and chemotactic cytokines such as IL-6, IL-10, HIF-alpha, VEGF, catalase, among others, would impair implantation, disfavoring fertility (Harada et al., 2016). Studies have also demonstrated endometrial receptor changes in patients with adenomyosis. The expression of molecules that convert androgen into estrogen in the endometrial environment of the adenomyosis carrier, such as the cytochrome P450 aromatase protein, would have a negative impact on both the clinical fertility rate and the IVF success rate. Studies also investigated whether therapy with GnRH agonists and danazol would have a positive impact on infertility, precisely because they reduce the expression of P450 aromatase in the endometrium (Harada et al., 2016; Mahajan et al., 2018). Other receptors responsible for the poor fertilization rates would be estrogen and progesterone receptors. IL-6, thanks to the intense and prolonged inflammatory process, is able to increase estrogen receptor expression in adenomyosis, similar to the P450 aromatase, a theory for the genesis of infertility, adding to the seemingly reduced expression of progesterone receptors that occurs in all layers of the junctional zone also contributing to a worse outcome (Harada et al., 2016).

Another issue that must be studied is the presence of local oxygen at the time of embryo implantation, and in this aspect, a higher oxygen rate imposes an excess of free radicals that damages the fertilized egg and interferes with the embryo development. In normal women, the concentration of substances such as nitric oxide synthase and superoxide dismutase, along with following the menstrual cycle, is generally lower than in patients with adenomyosis, favoring implantation in healthy women and hindering the embryo’s in vitro and in vivo development in the adenomyotic uterus (Harada et al., 2016).

Adenomyosis can also impair the implantation of the conceptus by providing the endometrial environment with a low expression of adhesive molecules, implantation markers and genetic alteration, as in the HOXA 10 gene, for embryonic development. Adhesive molecules, such as integrins, selectins and cadherins, expressed by the endometrium of patients without adenomyosis, and implantation markers, Leukemia inhibitory factor (LIF) for instance, must have their levels minimally elevated in the implantation window for embryo-endometrial interaction to occur. Low levels of these molecules in the adenomyotic endometrium would be responsible for patients who have failed IVF, even with good embryonic quality (Harada et al., 2016).

### Evidence from clinical studies

A causal relation between adenomyosis and infertility has been repeatedly suggested (Brosens et al. 2010; Campo et al., 2012; Sunkara & Khan, 2012; Tomassetti et al., 2013; Younes & Tulandi, 2017; Sharma et al., 2019), but definitive conclusions are still lacking. To determine whether adenomyosis negatively affects fertility, several researchers focused on women affected by the pathology that underwent IVF, since this model provides more accurate data about the influence of adenomyosis on embryo implantation (Vercellini et al., 2014). However, despite numerous theories justifying this association; studies investigating the association between adenomyosis and IVF outcomes are insufficient for a more accurate conclusion, in addition to presenting high heterogeneity and many biases. Furthermore, it is worth mentioning that there are no studies that investigate natural conception outcomes in women with adenomyosis.

According to a prospective study conducted by Benaglia et al. (2014) and Mavrelos et al. (2017), many of these studies are conflicting; while some have not reported a relevant statistical impact concerning pregnancy rates (Costello et al., 2011; Martínez-Conejero et al., 2011; Mijatovic et al., 2010), others found a significant negative association of adenomyosis and the likelihood of pregnancy and birth (Maubon et al., 2010; Ballester et al., 2012; Salim et al., 2012; Thalluri & Tremellen, 2012; Youm et al., 2011). Such studies report considerable differences of adenomyosis prevalence in the infertile population, ranging from 7% to 28% (Benaglia et al., 2014; Mavrelos et al., 2017). In addition, it is also important to emphasize that we found no studies associating adenomyosis in any way to negative conception outcomes in the literature.

The retrospective study by Mijatovic et al. (2010) aimed to establish the effects of adenomyosis on IVF/ICSI outcomes of infertile patients with endometriosis who were pretreated with a GnRH agonist for 3 months. No significant differences were found regarding pregnancy, implantation, miscarriage, ectopic pregnancy and ongoing pregnancy rates between the general population and the two subgroups (with adenomyosis versus without adenomyosis). Nevertheless, the study did not report a control group with patients with adenomyosis who were not pretreated with the GnRH agonist before in vitro fertilization/ICSI, which can generate a confounding factor. In turn, Martínez-Conejero et al. in 2011, sought to evaluate the effects of adenomyosis on gene expression in the endometrium, exclusively. For this, the group tested the successful implantation of donated oocytes, to eliminate the unfavorable outcomes related to the embryo, and pregnancy in women with the pathology, allowing the selective evaluation of uterine levels. In conclusion, they found that implantation is not affected by adenomyosis, but the higher rates of miscarriage associated with this condition lead to lower rates of pregnancy. Finally, the designs of the studies that did not report a relevant statistical impact concerning pregnancy rates in women with and without adenomyosis were different and had distinct objectives, not being able to conclude the real role of the pathology on embryo implantation.

On the other hand, in 2014, with the objective of more precisely defining the effect of uterine adenomyosis on the probability of pregnancy through IVF, Vercellini et al. (2014) conducted a systematic literature review and meta-analysis of data published in the last 15 years. The study analyzed published articles that compared the pregnancy rate, after IVF, of infertile women without adenomyosis with the pregnancy rate of those who had adenomyosis identified on TVUS and/or pelvic MRI. The authors concluded that adenomyosis is linked to 28% reduction in the probability of clinical pregnancy in infertile patients undergoing IVF with autologous oocytes; presenting a total relative risk of clinical pregnancy of 0.72 (95% confidence interval [CI], 0.55-0.95) and a risk of miscarriage of 2.12 (95% CI, 1.20-3.75) compared to controls (Vercellini et al., 2014).

In general, the harmful effects of adenomyosis on IVF seems to be related to reduced implantation rates, increased risk of early pregnancy loss and, as a result, a decrease in live births rates (Younes & Tulandi, 2017; Harada et al., 2019), which could be directly related to the anatomo-physiopathological changes generated by adenomyosis in the female genital tract, including impaired utero-tubal transport, reduced sperm function due to high nitric oxide levels in the uterine cavity, altered uterine contractility, altered endometrial capillary density, excessive angiogenesis mediator secretion, reduced expression of implantation markers, inadequate decidual reaction owing to the overexpression of P450 aromatase, which alters the estrogen/progesterone balance in the secretory phase of the cycle, and many others (Mavrelos et al., 2017).

Moreover, when studying adenomyosis in women who have undergone IVF, the viability of the pregnancy must be taken into account. According to the review conducted by Dueholm (2017), seven studies reported miscarriage rates in IVF studies; 32% occurred in women with adenomyosis, 14% in women without adenomyosis, with a common RR of 2.12, 95% CI 1,20-3,75. Thus, there was an association between adenomyosis and spontaneous abortion. Furthermore, according to the author, two studies (Juang et al., 2007; Mochimaru et al., 2015) evaluated the relation between adenomyosis and premature birth, and reported an increased risk of the latter linked to the pathology in question (Duelhom, 2017); data that corroborates the results were found later by Horton et al. (2019) and Porpora et al. (2020).

Still regarding the unfavorable pregnancy outcomes, a recent retrospective study by Stanekova et al. (2018) was the first to show that adenomyosis is associated with an increase in early pregnancy loss, regardless of maternal age and genetic status of embryos. Even though several previous studies also reported an increase in miscarriage rates in women with adenomyosis, many did not adequately control confounding factors for pregnancy loss risk, such as older age and higher BMI (Younes & Tulandi, 2017). Stanekova’s study was the only one in which solely high quality euploid embryo transfers were submitted to genetic analysis to control the risk of embryo aneuploidy related to maternal age, hence strengthening the direct causal link between adenomyosis and early pregnancy loss. This protocol is similar to the alternative approach taken by Martinez-Conejero et al. (2011), mentioned above, in which they investigated adenomyosis using embryos created from young oocyte donors. It should be noted that both approaches are consistent with the premise that the adenomyotic uterus provides a dysfunctional environment for pregnancy maintenance, in addition to a potentially hostile environment for initial implantation events; however, it is extremely important to emphasize the need for studies that analyze embryo euploidy prior to implantation, in order to rule out genetic abnormalities as causes of IVF failure.

Furthermore, the differences of adenomyosis prevalence and its impact on IVF may be directly related to the use of distinct criteria and diagnostic methods to detect adenomyosis, therefore constituting an important bias in studies that research the possible correlation between the pathology and infertility, as warned by Gordts et al. (2008) and later reaffirmed by Chapron et al. (2020). According to Meredith et al. (2009) and Maheshwari et al. (2012), transvaginal ultrasound (TVUS) and magnetic resonance imaging (MRI) are reliable diagnostic modalities, although MRI may be slightly more advantageous (Champaneria et al., 2010). As previously described, the suggestive characteristics of adenomyosis, in TVUS, are asymmetric thickening of the anterior and posterior myometrium, abnormal endometrial-myometrial interface (EMI), heterogeneous hypoechoic areas on the myometrium, all together with myometrial anechoic lacunae. For many authors, the presence of three or more ultrasound characteristics are suggestive of adenomyosis, which would justify the use of this tool for adequate screening according to Naftalin et al. (2012) and Duelholm, 2017. However, studies using different combinations of these criteria are found in the literature, on top of considering them in a binary way that is, adenomyosis present or absent, being a source of heterogeneity in the literature (Mavrelos et al., 2017). An example of the importance of standardizing diagnostic criteria is demonstrated by the data found by Mavrelos et al. (2017) and Sharma et al. (2019), which directly correlated the severity of adenomyosis with an increased chance of IVF failure, a higher rate of pregnancy loss, and diminished live birth rates regardless of the woman’s age and ovarian reserve; that is, when women have four or more characteristics of adenomyosis on examination, the likelihood of pregnancy is halved, possibly representing a more severe form of the disease, since milder forms, presenting less criteria, had a limited impact on IVF success (Mavrelos et al., 2017; Sharma et al., 2019). In addition, we noticed that most studies do not relate the differential impact of each characteristic found to the diagnosis. In fact, while the heterogeneous classification of the disease in studies utilizing USTV could lead to the inclusion of some women with milder forms of adenomyosis in the adenomyosis group, resulting in an underestimation of the association between adenomyosis and the IVF outcomes; the use of MRI based exclusively on different cut-off points for the junctional zone (JZ) thickness can lead to the selection of different study groups, hence limiting them (Vercellini et al., 2014).

For infertility investigative purposes, one must also take into account the ample evidence that adenomyosis often coexists with other gynecological diseases, such as uterine fibroids and, especially, endometriosis, pathologies that are commonly associated with pelvic pain and dysmenorrhea. Li et al. (2014) conducted, a retrospective review, in which 710 pre-menopausal women with adenomyosis were submitted to hysterectomy, detecting that 343 (48.3%) had adenomyosis alone, 158 (22.3%) adenomyosis and endometriosis, 129 (18.2%) adenomyosis and uterine fibroids, and 80 (11.3%) the three conditions combined (Chapron et al., 2020). On the other hand, the presence of endometriosis in patients with adenomyosis has been reported in 80.6% of the cases, while adenomyosis was present in 79% of the patients with MRI-diagnosed endometriosis, with a clear relation between the thickness of the JZ and the severity of endometriosis (Kunz et al., 2005).

Given this, the proportion of women with both diseases remains controversial and the diagnostic criteria also remain an obstacle (Kunz et al., 2005; Bazot et al., 2006). As endometriosis has been correlated with subfertility and the reduced probability of conceiving after assisted reproductive technology (ART), it is extremely important to carry out studies that investigate IVF outcomes in women with endometriosis only, adenomyosis only, and those with both pathologies. Sharma et al. (2019) did this in a retrospective study, when they evaluated, for the first time, the effects of adenomyosis with and without endometriosis on ART results. The group analysis found a lower clinical pregnancy rate, a higher spontaneous abortion rate, a lower live births rate, and a higher rate of pregnancy complications in patients with adenomyosis alone or in the presence of endometriosis, when compared to women with endometriosis or infertility only related to the tubal factor (control), suggesting a negative effect of adenomyosis on the overall IVF outcomes, corroborating data already published by Landi et al. (2008), Costello et al. (2011), and Salim et al. (2012), and that the chances of miscarriage are higher in adenomyosis, regardless of the quality of the oocyte or embryo, as shown by Martínez-Conejero et al. (2011) and Vercellini et al., 2014. However, based on the analysis of available evidence, it is still difficult to understand to what extent IVF failure is due to the presence of endometriosis or adenomyosis, further reinforcing the need for uniformity and standardization of diagnostic imaging criteria.

Another incongruity when comparing the studies is whether treatment prior to IVF had been performed. As already mentioned, adenomyosis in infertile women may be treated surgically or clinically with the use of GnRH agonist (GnRHa); however, achieving a balance between removing the adenomyosis completely and preserving the normal uterine contour during pregnancy can be an obstacle. Another important issue that must be considered is the elevated risk of uterine rupture during pregnancy or childbirth after surgical treatment, using a gonadotropin-releasing hormone (GnRH) agonist preferable over selective progesterone receptor modulators (SPRMs), such as ulipristal acetate (UPA) (Park et al., 2016; Donnez & Donnez, 2020). Given that GnRH receptors are found on adenomyotic lesions, GnRH agonists are used for medical treatment and have a direct antiproliferative effect on the myometrium. In addition, they can markedly reduce the inflammatory reaction and angiogenesis, as well as significantly inducing apoptosis in adenomyosis-derived tissues. Besides its direct antiproliferative effect within the myometrium, a hypoestrogenic effect may be involved in the adenomyotic lesion regression, uterine size reduction and symptom relief. In a retrospective study carried out by Park et al. (2016), three different groups were compared based on the IVF strategy employed: fresh embryo transfer cycles, fresh embryo transfer cycles including GnRH agonist pretreatment, and frozen-thawed embryo transfer (FET) cycles with GnRH agonist pretreatment. When comparing the fresh embryo using cycles, the GnRH agonist pretreatment was not significant, which, in turn, was noteworthy when analyzing the frozen-thawed embryo using cycles, demonstrating a greater potential to develop a successful pregnancy. Such results align with those demonstrated by Mijatovic et al. (2010), Tremellen & Russell (2011), Costello et al. (2011) and Niu et al. (2013) and contradict those presented by Sharma et al. (2019), which justifies, once again, the need for further studies to guide a standard clinical treatment.

Finally, although the vast majority of studies found in the literature investigate the impacts of adenomyosis on fertility in women undergoing IVF, a recent cross-sectional study by Hashim et al. (2020) focused on analyzing the prevalence of this pathology in a population of infertile young people. To this end, the study was carried out with 320 women under the age of 41 who attended an infertility clinic and who had not previously been diagnosed with adenomyosis. They were screened for the disease by looking for adenomyosis markers using two-dimensional transvaginal ultrasound (2D-TVUS), and later confirmed by magnetic resonance imaging (MRI). Adenomyosis was detected by 2D TVUS in 7.5% of cases and confirmed by MRI in 6.6%, the intrinsic form being the most commonly found. In addition, the study found that women with adenomyosis had a higher average age, a higher BMI, more dysmenorrhea complaints and a higher ovarian endometriomas (a marker of severe endometriosis) incidence than those without adenomyosis. Therefore, the study stresses that specialists in the initial management of patients investigating infertility should consider adenomyosis. Thus, the hypothesis that adenomyosis may cause changes to the uterine environment that hinder embryonic implantation in natural conceptions and that, if present, may also influence if the patient is submitted to IVF.

Analyzing all the exposed data, it is evident that there are a large number of biases present in the few studies carried out, which, in addition to being only observational, consider different characteristics for selection, such as: studied sample size, age differences between patients, socioeconomic differences, infertility duration, degree of adenomyosis, coexistence of other pelvic disorders, previous treatment protocol; quality, number and stage of transferred embryos, number of in vitro fertilization cycles performed and, above all, modality used to diagnose the adenomyosis.

Therefore, the need to conduct randomized, large-scale clinical studies, with well-defined and standardized selection criteria to conclusively associate adenomyosis with a poor reproductive outcome is undeniable.

## CONCLUSION

Adenomyosis appears to have adverse effects on in vitro fertilization results, clinical pregnancy rates, live birth rates and pregnancy loss rates. Establishing a standardized diagnostic protocol is of great relevance, seeing that screening for adenomyosis must be considered before assisted reproductive treatment, both for counseling women with adenomyosis and for elucidating the prognosis. Even though MRI can theoretically provide better information than TVUS, the latter should be preferred for screening, since it has greater availability and low cost, leaving MRI for specific situations.

Moreover, further research, eliminating all risk factors associated with embryo aneuploidy are needed. These risks are, for instance, maternal age and the potential consequences of adenomyosis, regarding great obstetrical syndromes, such as miscarriage, premature birth, intrauterine growth restriction, preeclampsia and obstetrical hemorrhages

Thus, the evidence is impaired by the poor quality of the studies, the lack of strict imaging diagnosis, and the absence of a classification according to the diseases extent. The selection of ideal evidence-based treatment options for adenomyosis in fertility clinics is difficult, due to the lack of evidence that there is a relation between fertility and the degree and composition of adenomyosis, reinforcing, once again, the need for standardized studies.
